# Material Flow in Infeed Rotary Swaging of Tubes

**DOI:** 10.3390/ma14010058

**Published:** 2020-12-24

**Authors:** Yang Liu, Jing Liu, Marius Herrmann, Christian Schenck, Bernd Kuhfuss

**Affiliations:** 1 Bremen Institute for Mechanical Engineering–bime, Badgasteiner Str. 1, 28359 Bremen, Germany; herrmann@bime.de (M.H.); schenck@bime.de (C.S.); kuhfuss@bime.de (B.K.); 2 University of Bremen, Bibliothekstraße 1, 28359 Bremen, Germany; 3 Key Laboratory of Materials Processing Engineering, School of Material Science and Engineering, Xi’an Shiyou University, Xi’an 710065, China; jingliu@xsyu.edu.cn; 4 MAPEX Center for Materials and Processing, Am Biologischen Garten 2, 28359 Bremen, Germany

**Keywords:** metal forming, cold forging, combined hardening

## Abstract

Rotary swaging is an incremental metal forming process widely used to reduce the cross–section of parts. For tubular parts, the final wall thickness also changes during the process. The lubricant condition is a factor, which affects these geometry changes. Beneath the change of the geometry, the complex material flow during the process determines the final geometry and the mechanical properties. Therefore, with a thorough insight into the material flow, it could be understood how to control it in order to achieve desired properties. Producing tubes with uniform outer diameter and changing inner profiles is an application of this method. Furthermore, applying this method, different local cold hardening could be achieved by different total strain. In this study, the dependency of the material flow on the lubrication conditions was investigated. Simulations with combined hardening material models were verified by the change of the wall thickness of tubes. It was found that friction condition significantly influences the back shifting of the workpiece and the elongation caused by each stroke. Results from simulations and experiments showed that a certain lubricant condition leads to the highest axial elongation of the workpiece.

## 1. Introduction

Rotary swaging is an incremental precision forging process, which is used to reduce the cross–section of parts with full or hollow sections [1]. In the process, the diameter of the workpiece is reduced and the material flows simultaneously in axial and radial directions. As an incremental forming process, the reduction of the section is finished in many strokes. With every incremental forming step, just a small amount of material is deformed. This leads to advantages like small deformation loads and long die life [2]. With the work hardening effect, the strength of the workpiece can be improved, which makes it possible to replace some expensive materials with cheaper materials [3,4]. Additionally, after the process, the surface condition could be improved with a lower roughness than the initial state [5]. Except for widely used structural steel and soft materials like aluminum, this process is able to deform metals like titanium, magnesium, and tungsten [6]. Due to these advantages, rotary swaging has been widely used in industry [7]. In the rotary swaging system, there are at least two dies in the swaging head. They are arranged symmetrically around the centre axis. In the process, the dies rotate together with the swaging head while they perform a radial oscillation toward the axis. In the infeed rotary swaging process, the workpiece is fed into the dies with the axial feeding velocity v_f_.

**Figure 1:**
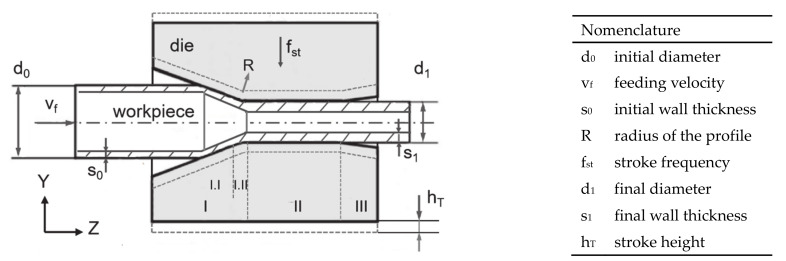
Principle of infeed rotary swaging of tube without mandrel.

The principle of infeed rotary swaging of a tube without mandrel is illustrated in Figure 1. The dies feature three functional areas: the reduction zone (I), where the main plastic deformation happens; the calibration zone (II), where the final diameter of the workpiece is precisely finished; and the exit zone (III), where the part is smoothly unloaded. In the reduction zone, according to the geometry features, it was divided into two areas: in I.I (forging zone), the profile of the 2D sketch of the die is straight; in I.II (transition zone), the die featured an arched profile with a radius (R) equals 30 mm. In the process, the workpiece is fed into the dies with feeding velocity (v_f_), the dies are striking the workpiece with a certain frequency (f_st_) and stroke height (h_T_). Determined by these parameters, the feed per stroke (l_st_) thus the length of the workpiece fed in each stroke is also a quasi–constant value. The tube features the initial diameter (d_0_), the initial wall thickness (s_0_) before forming, the final diameter (d_1_), and the final wall thickness (s_1_) after the process.

During the forming process, along with the infeed motion of the workpiece, zone I is gradually filled with increased material volume to be deformed in each stroke. When the tip of the workpiece reaches zone II, the contact length between the workpiece and dies is still not constant, which affects the friction force. After zone II is filled and the material reaches zone III, in each stroke the contact length between the workpiece and the dies is assumed to be constant and the friction condition does not change anymore. The subsequent analysis based on simulations and experiments is considered in this quasi–stationary phase.

In the process, the local material experiences complex deformation, for example in one stroke it could be axially compressed and in the next stroke, it is axially stretched. This depends on the relative location of the local material according to the die [8]. Therefore, Bauschinger effect due to cyclic loading should be considered in the material model. However, many publications used the basic material model based isotropic hardening law such as Hollomon law [9,10,11], Johnson–Cook model, or Ludwik model [12]. With these models, the stress tends to be overestimated under cyclic loading [13]. In consequence, there could be deviation of the plastic strain and the final residual stress [14]. For a precise finite element (FE) model of rotary swaging, the material model based on combined hardening law is preferred. Additionally, with the rising demand of reducing costs and protecting the environment, dry rotary swaging has been analyzed by using coated and/or structured tools to control the friction conditions in the process. With the possibility of achieving different friction conditions, the forming result could be changed in terms of geometry, stress state, and so on, which has not been investigated thoroughly. Furthermore, without lubricant, material from the workpiece could adhere to the die surface. Another potential problem is the damage to the coating due to relative material flow on the contact pair.

Rotary swaging has been widely investigated in terms of theoretical calculation, simulation and experiment. It was found that the deformation is inhomogeneous [15], the material on the ends of the contact shows local highest elongation. Wu et al. [16] investigated the inhomogeneity (as a reason of final convex or concave shape formed on the tip of the workpiece) of axial material flow in rotary swaging process and verified the model by comparison with published experimental data. They found that the shape was formed in the preforging state. To reduce the inhomogeneity of axial material flow, a lower relative radial reduction and higher feed per stroke were preferred. Sanjari et al. [17] investigated the strain field of tube rotary swaging with mandrel, the FE simulation results were compared with the results of microhardness measurements. The minimum and maximum strain appeared at the inner and middle of the radius respectively. The inhomogeneity could be reduced to a minimum with convex shape dies. Moumi et al. [18] investigated the neutral plan of infeed rotary swaging based on a 2D–axisymmetric FE model. The neutral plane was found to be varying during the process. For softer material like aluminum, the locations of neutral plane were closer to the deformed part than for harder material like steel. To visualize the axial material flow in eccentric thread swaging, Ishkina et al. [19] developed a special die on which a small cam was manufactured, they found that the pitch of the resulting thread is not constant over the feeding direction, which means an increasing fluctuation of the axial material flow. In the former research [20], rod workpiece with different friction coefficient was investigated in rotary swaging, it was found that the ¼ radial material from the outer surface is sensitive to friction condition, and that on the center of the axis and the ends of the reduction, the local material flow is most active. However, the research was only based on rod workpiece and only the equivalent plastic strain was investigated instead of the plastic strain components on different directions.

As found in former research, cyclic loading was often neglected in FE models. Additionally, with the development of dry forming technology, the process could be realized with complex friction conditions. With this background, the features of material flow had not been much investigated. On the contact surface, with local high normal stress and material flow, wear or adhesion of the material on the coating surface is becoming the main limitations of the forming performance and the lifetime. Thorough knowledge of the influence of friction condition on plastic deformation is still not achieved, based on which the material flow could be better controlled and at last lead to the optimized process. With the knowledge of the material flow on the contact pair, these issues will also be benefited. In this study, infeed rotary swaging process of tubes made of steel alloy (1.0308) was investigated in both FE model and experiments, in the FE model, an isotropic/kinematic hardening model is developed based on the cyclic test. The features of geometry and plastic strain component were investigated in the complete process and also in a detailed single stroke. The influence of friction condition is thoroughly investigated and the findings were verified by the experiments.

## 2. Methods

### 2.1. FE Model

**Figure 2:**
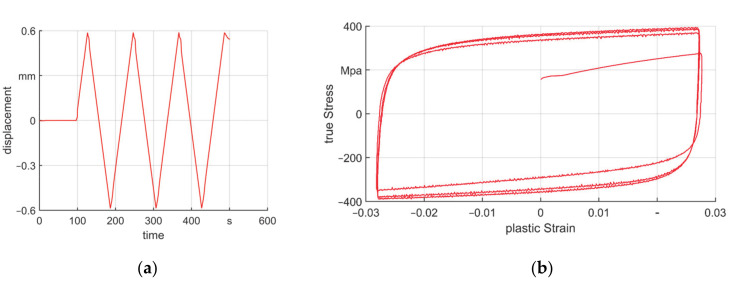
Cyclic test, (**a**) loading and unloading curve and (**b**) stress–strain curve of the material.

The FE model was built in ABAQUS (version 6.13) based on assumptions in [8]. A 2D axisymmetric model was generated and explicit time integration was used. The workpiece was set as deformable with a length of 300 mm and, its diameter was reduced from Ø 20 to Ø 15 mm in the process. The initial wall thickness values were: s_0_ equals 3, 4, and 5 mm (T3, T4, and T5). The material was steel alloy (1.0308) (Roland Stahl, Bremen, Germany) with Young’s modulus equaled 210 GPa and Poisson’s ratio equaled 0.3. A constitutive model with combined hardening law was used to include the Bauschinger effect due to cyclic loading. The cyclic test experiment was carried out, in which the strain rate equaled 0.0012/s. The loading and unloading curve is shown in Figure 2a and the stress–strain curve is shown in Figure 2b. The parameters of the model are shown in Table 1 according to the method introduced in reference [21]. On the workpiece, a square–shaped CAX4RT mesh was used over a length of 80 mm, which is the part to be swaged. In this part, the radial mesh number was 9, 11, and 13 for T3, T4, and T5 to achieve a trade–off between avoiding hourglass phenomenon and calculation cost [22]. Regions far away from the deformation area were meshed with much bigger meshes to reduce the simulation cost.

The die was set as an analytical rigid body so that meshing was not needed. The die angle was 10°. The length of the calibration zone was 20 mm. The friction condition on the contact area between the workpiece and dies was assumed to be constant and follow coulomb law, however, a limit was set as yield shear stress [23], which is usually estimated as σ_s_/$\sqrt{3}$. The friction coefficients in this study were set in the range of 0.1–0.4 with an interval of 0.05 to compare different die surfaces effects and lubrication conditions, which there are in the real–world applications. The lowest value of μ was set as 0.1 because using a coating such as diamond like carbon (DLC) or chromium nitride (CrN) combined with lubricant, the friction coefficient can reach 0.1, and in dry condition, it can reach 0.4 [24], which is also used in cold forging simulations [25]. According to the self–locking theory, for this die angle there is a certain value of μ, which equals tan (10°) ≈ 0.18. So, this value of μ was also considered in the simulation.

**Figure 3:**
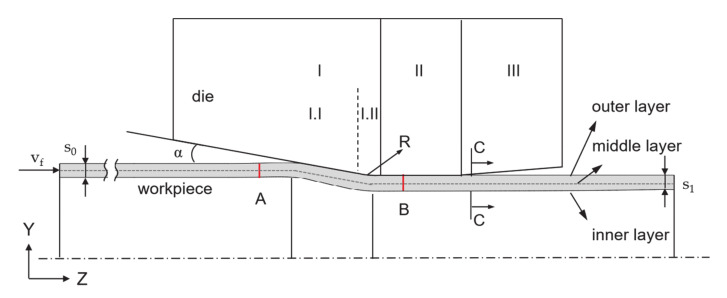
Featured areas and layers in the FE model.

During the process, the feeding motion of the workpiece along Z–axis with feed per stroke (l_st_), and the striking motion of the die along the Y–axis with a stroke height h_T_ = 1 mm, were controlled by time–displacement tables. The state at the end of the process is shown in Figure 3. The area between positions A and B were selected as a representative area covering both the material out of the contact region and inside calibration with deformed geometry. The final state was taken at section C–C in the exit zone, which is 2 mm behind the boundary between zones II and III. The wall thickness of the workpiece at the C–C section was measured according to the method introduced later.

**Figure 4:**
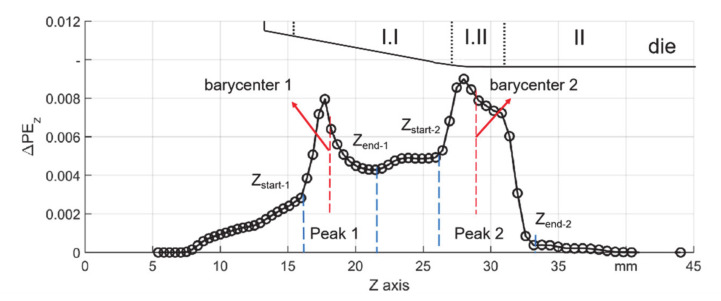
Description of the two local ΔPE_z_ peaks and the barycenter.

To analyze the simulation result, plastic strain components were introduced, according to the coordinates, the plastic strain along the Z–axis (axial direction) was named as PE_z_ while that along the Y–axis (radial direction) was named as PE_y_. These two components represent the strain state of the local material on axial and radial directions respectively. The increment of plastic strain, named as ΔPE_z_ and ΔPE_y_, was studied to quantify the difference in one stroke. ΔPE_z_ contributes to the overall elongation whereas ΔPE_y_ contributes to the final wall thickness. As the former research showed, there are local positions where the material flow is the most active [15,20], but the features of the active areas and the influence of the friction condition were not thoroughly investigated, which could further affect the die life. Local peaks were defined to describe the feature of the ΔPE_z_ (Figure 4). The two peaks are generated by the contact between the die and the workpiece. They are correlated to the locations where the geometry of the die changes, i.e., the first contact in zone I.I and zone I.II. The peaks (Peak 1 and Peak 2 within the blue dash lines) were found according to the following principles: the start position of the peak was the last node after which the deviation is obviously higher than the nodes before, the Z coordinate of the start points were named as Z_start–1_ and Z_start–2_, the end position of Peak 2 is the same as (Z_end–2_); the end position of Peak 1 was chosen at the position with the local lowest value of ΔPE_z_. The representing height of the two peaks (mean ΔPE_z_) was calculated by the mean value of ΔPE_z_ of all nodes within the boundaries Z_start_ to Z_end_. The coordinates of barycenter were calculated by finding the position where half of the representing height was reached.

### 2.2. Experiments

**Figure 5:**
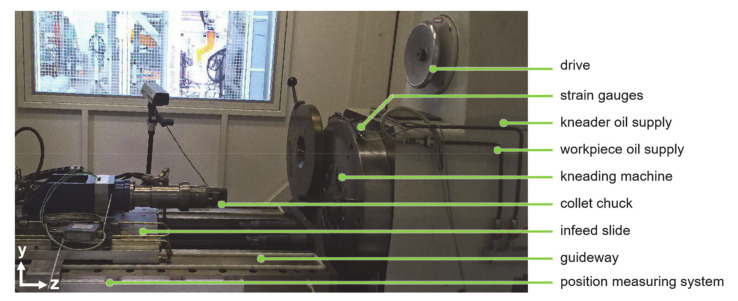
Rotary swaging machine.

**Figure 6:**
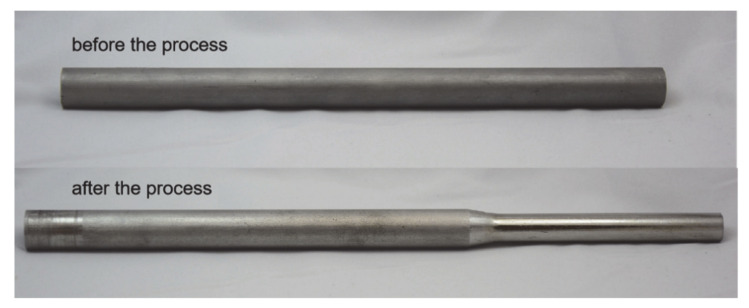
Workpiece before and after rotary swaging.

The rotary swaging machine (Felss Holding GmbH, Königsbach–Stein, Germany) is shown in Figure 5. Oil supply can be turned off for dry forming. In the process, the workpiece is held by the collet chuck and fed (along the z direction) into the kneading machine, after a certain feeding length, the deformed workpiece is pulled out to the original position. The tubes with different s_0_ were made of rods with a diameter of 20 mm and a length of 300 mm by drilling. Depth of drilling was 80 mm, which was enough to achieve several strokes after the calibration zone is filled (quasi–static state). For each setting, the experiment was repeated three times. The workpiece before and after the process is shown in Figure 6.

Two types of dies were used to investigate different friction conditions. The first type is commercially available die (material ASP2013, Felss Holding GmbH, Königsbach–Stein, Germany), which featured a tungsten carbide layer in the reduction zone (conventional dies). The second one is (material 1.2379, Roland Stahl, Bremen, Germany) featured with the same geometry but an a–C:H coating on the functional areas, which is in contact with the workpiece (coated dies) [26]. For lubrication, the Condocut KNR 22 oil (FRIEDRICH SCHARR KG, Stuttgart, Germany) was used. For the dry forming process, the workpiece and dies were thoroughly cleaned before each experiment. During the experiments, three friction conditions were evaluated from low to high friction for: coated dies with oil, coated dies without oil, and conventional dies without oil respectively. In the forming process, the tubes were fed into the machine with a constant feeding velocity equaled to 500 mm/min, the striking frequency f_st_ = 37.5 Hz, and the stroke height h_T_ equaled 1 mm. The feed per stroke value was calculated as 0.22 mm. After an infeed length of 80 mm the workpieces were directly pulled back. To measure the wall thickness of the sample as a validation, two methods were used.

**Figure 7:**
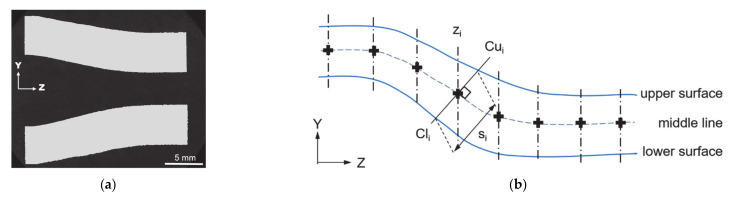
(**a**) Longitude section sample and (**b**) method to calculate the wall thickness between two boundaries.

The first one is based on the longitude section (Figure 7) of T5 showing the development of the wall thickness. This part covers the initial and final state of the workpiece, the calculation is illustrated in Figure 7b. The local wall thickness s_i_ is defined as the distance between the crossing points Cu_i_ and Cl_i_ of the upper and lower surface with the line orthogonal to the middle line between the surfaces in position z_i_. The length of the longitudinal section was made from the first contact to the beginning of the calibration zone, i.e., 15–33 mm in Figure 4. This method is also used for the wall thickness calculation in simulated results.

**Figure 8:**
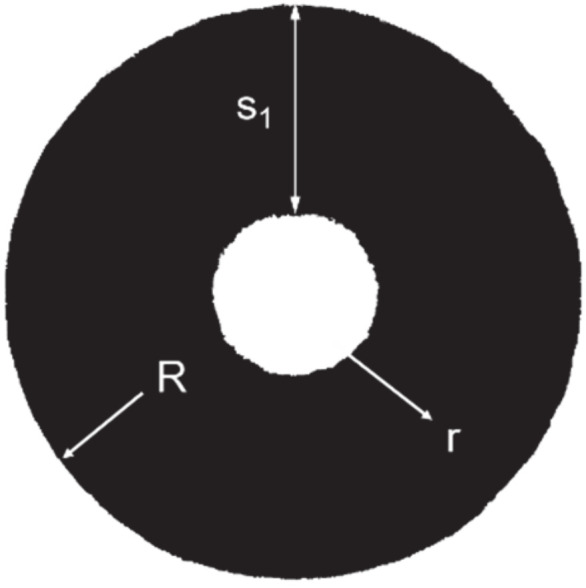
Method to calculate equivalent wall thickness of the cross–section.

The second method to determine the final wall thickness was made on the cross–section at the same position as section C–C (Figure 3). Figure 8 is an example of the sample. The wall thickness was calculated by means of digital image processing. Therefore, the cross–sections were binarized. The outer profile of the sample could be fitted with a circle in the post–processing software of the microscope, thus the value of R can be read directly. Then, numbers of the black pixels of the sample with black color (N_k_) and of the bore with white color (N_w_) were counted. The equivalent radius r of the inner profile and the equivalent wall thickness s_1_ could be calculated as Equations (1)–(3). Then, the FE model was validated by comparing the final wall thickness values.

(1) $$\frac{\pi r^{2}}{\pi R^{2}}=\frac{N_{w}}{N_{w}+N_{k}}$$

(2) $$r=R\times \sqrt{\frac{N_{w}}{N_{w}+N_{b}}}$$

(3) $$s_{1}=R-r$$

## 3. Results of Simulation

During the simulation process, the sum of Internal Energy is marked as ALLIE while the sum of Kinetic Energy is marked as ALLKE. To verify if hourglass is nonegligible in the calculation, the values of ALLKE/ALLIE (the ratio of kinetic energy to inner energy) were monitored. It was found that after the first several strokes, i.e., when the deformed material reached calibration zone, they were always lower than 5%, which is much lower than the common threshold 10% [27], thus the models were stable. Then the geometry and plastic strain features were investigated during the process.

### 3.1. Geometry Features

**Figure 9:**
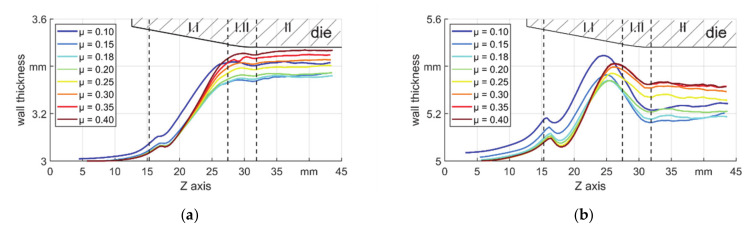
Wall thickness developments, (**a**) s_0_ = 3 mm and (**b**) s_0_ = 5 mm.

In rotary swaging, the workpiece ends with a longer length and changed wall thickness. The development of wall thickness is shown in Figure 9. Some features can be found as follows:

1. Before the contact of die and workpiece (left of zone I.I)–the wall thickness of the workpiece shows an increase especially for low μ;
2. The wall thickness increased in zone I.I, but on the locations with a changing profile of the die, i.e., the location of first contact (zone I.I) and the boundary between zones I.I and I.II, the wall thickness tended to reduce. This was more obvious in thicker tubes;
3. The final wall thickness changed depending on friction coefficient. As could be seen, there was a certain friction coefficient (μ = 0.18), which led to the thinnest wall (longest workpiece).

**Figure 10:**
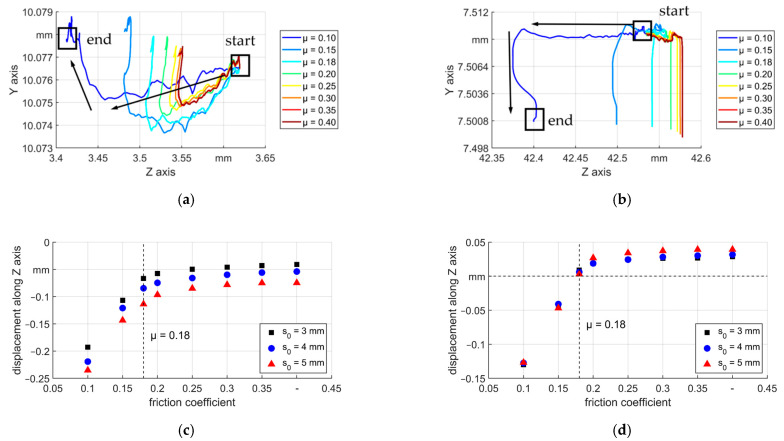
Routes and axial displacements of boundaries A and B, (**a**) routes of A, s_0_ = 5 mm, (**b**) routes of B, s_0_ = 5 mm, (**c**) axial displacement of A, and (**d**) axial displacement of B.

An increased wall thickness together with an increased outer diameter (shown later) before the reduction zone shows the existence of backward material flow. To have a clear view of the back and forward motion, the movement of the boundaries A and B during a stroke was shown in Figure 10a,b. The examples were from the results of T5, the curves showing the movement of the boundaries with μ = 0.1 are illustrated by arrows. The displacement of boundary A is shown in Figure 10c. It could be seen that the distance was higher for thicker tubes due to higher volume to be deformed in the radial direction, which led to higher backward material flow. Additionally, it can be found that the displacement reduced with higher μ, so the backward material flow was reduced. In Figure 10d the displacement of the boundary B is shown, in which different features could be investigated as: when μ was lower than 0.18, the boundary moved backwards and while it was higher than 0.18, the boundary moved forward. The friction coefficient over the self–locking condition reduced the backward motion of the deformation area. As the friction coefficient increased, the change of the displacement was less obvious. This can be explained by the friction model with a limit. For the material in the deformation area, i.e., forging and transition zone where the normal stress was quite high, the limit could be reached and higher values of μ caused little differences. However, in the calibration zone, the forward material flow will always be restrained by friction resistance.

**Figure 11:**
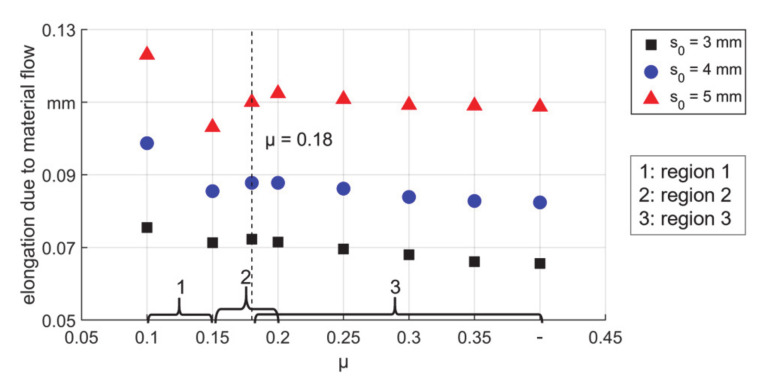
Elongation between A and B after one stroke due to material flow.

The distance between A and B when the die was completely closed consisted of both elastic and plastic elongation of the deformed area. Since the two boundaries were selected beyond the deformation area, in two consecutive strokes the elastic elongation between A and B could be treated as a constant. Then the difference of the distance between them shows qualitatively the plastic elongation of the deformed area of the workpiece, see Figure 11. Three regions (1, 2, and 3 in Figure 11) according to the friction coefficient could be identified. The first region was from μ = 0.1 to μ = 0.15: when μ = 0.1, the workpiece tended to be pushed back from the dies (backwards shifting), so the highest backward motion could be seen (compare also Figure 10c, d). Furthermore, a surface with the lowest μ means the lowest resistance to material flow along with both directions. In Figure 9, it could be seen that the wall thickness before entering the deformation area reached the highest value for μ equals 0.1. This means more material was fed into the dies in each stroke, so the values of plastic elongation of all the tubes with μ equals 0.1 were the highest. When μ increased to 0.15, the backward shifting was reduced and the backward material flow. Additionally, the resistance for forwarding material flow was higher and the increased wall thickness was also less, so the values of elongation were lower. Second region: from μ = 0.15 to 0.18 (T5) or 0.2 (T3 and T4) depending on the wall thickness, the back shifting was reduced to zero (compare Figure 10d), so the workpiece was held tightly by the dies, which benefited the reduction of the cross–section and led to higher elongation. Third region: higher than μ = 0.18 (T5) or 0.2 (T3 and T4) according to different wall thicknesses. In this region, with even higher values of μ, self–locking always existed in the reduction zone. The axial material flow was restrained more in the calibration zone, which led to less elongation.

**Figure 12:**
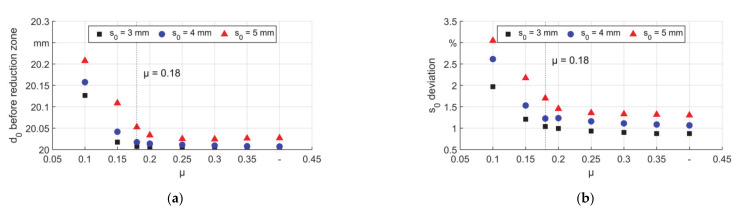
Geometrical information before material enters the reduction zone, (**a**) diameter and (**b**) thickness.

As a result of the backward material flow, the outer diameter and the percentage of increment in wall thickness before the contact increased (Figure 12). It is seen that low μ led to much higher diameter than those for higher μ values, after the self–locking, the diameter did not change obviously. For thick tubes, due to higher volume and thus higher forces of deformed material in each stroke, the backward material flow was higher compared to thin tubes. This led to a bigger initial diameter and thicker walls. In Figure 12b it can be seen that for T5, the wall thickness before the contact increased up to 3% with μ equals 0.1. Thus very low friction coefficient in the process could lead to lower precision of the product. It should also be noticed that there was always an increased diameter before the material entered the reduction zone, thus there was always an upsetting. During the stroke, the workpiece was always fed towards the dies, which made the deformation a combination of swaging and upsetting.

According to the findings, considering both precision and maximum elongation of the process, the friction condition should be neither too high nor too low. Although the elongation was the highest value for the lowest friction coefficient, the backward shifting and material flow was also the highest, which could bring a high load to the feeding system. The friction coefficient should not be too low because it can lead to a lower precision of the wall thickness. Otherwise, for higher friction coefficients, the elongation starts to get lower and the wall thickens at the final product. Furthermore, higher friction can lead to enlarged wear phenomena and worse surface quality. Thus the friction should be around the self–locking value.

### 3.2. Field Features in One Stroke

**Figure 13:**
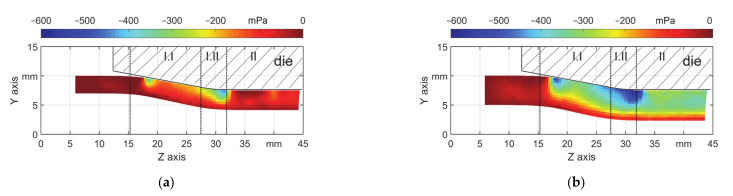
Radial stress field (S_11_) for μ = 0.18, (**a**) s_0_ = 3 mm and (**b**) s_0_ = 5 mm.

**Figure 14:**
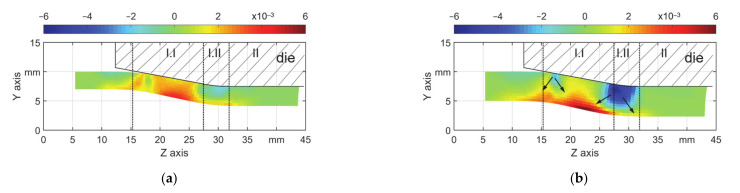
Radial plastic strain field (ΔPE_y_) for μ = 0.18, (**a**) s_0_ = 3 mm and (**b**) s_0_ = 5 mm.

The change of the geometry of the workpiece during a plastic forming process is due to the plastic deformation. The plastic strain components should be investigated to better understand the geometry features. The local geometry changes described in Chapter 3.1 can be explained by radial stress component (S_11_). For both T3 and T5 with μ = 0.18, the field of S_11_ at the end of a stroke are shown in Figure 13a, b. The field for other friction coefficients shows similar features. It is seen there is a stress concentration (local lowest S_11_) in the workpiece. Especially in the area of the transition zone (I.II), the material on those locations is radially compressed. This stress state is comparable with that of free bending, which on the area of the contact of the die there is compression stress and on the down surface of the workpiece there is tensile stress. As a result of the stress state, accordingly, ΔPE_y_ in Figure 14 shows the lowest values. Thus, there is an obvious radial material flow towards the inner surface (arrows in Figure 14b), thus the material on the inner surface shows the maximum stretching near the middle of the point of first contact and the boundary between zones I.I and I.II.

**Figure 15:**
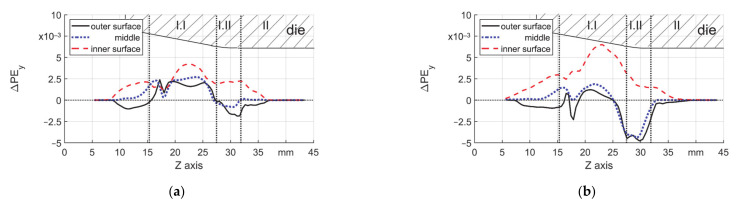
ΔPE_y_ on three layers, μ = 0.18, (**a**) s_0_ = 3 mm and (**b**) s_0_ = 5 mm.

To have a quantitative view, the distribution of ΔPE_y_ (radial direction) on the three featured layers, outer and inner surface and middle, are shown in Figure 15a,b. The values on the other layers were in the same range as in those three layers. The material on the outer surface and nearby experienced cyclic loading: compression, stretching, and again compression. The middle layer experienced much less cyclic loading, whereas the material in the inner surface only got stretched. Due to the stress concentration around z = 17 and 30 mm, the summation of radial ΔPE_y_ values at those locations show the lowest local values for all of the tubes, which indicates the lowest increase of the wall thickness (Figure 15a) for T3 and decrease of the wall thickness (Figure 15b) for T5. This explains the second feature in Chapter 3.1 that the wall thickness at the beginning of reduction zone and in the transition zone shows a less increment for T3 (Figure 9a) and an obvious reduction for T5 (Figure 9b).

**Figure 16:**
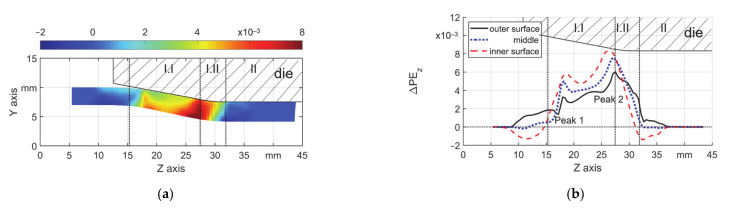
ΔPE_z_ in one single stroke, s_0_ = 3 mm, μ = 0.18, (**a**) field and (**b**) three featured layers.

To explain the plastic elongation in one stroke, the field of ΔPE_z_ (axial direction) was investigated. As an example, the field of ΔPE_z_ (T3, μ = 0.18) is shown in Figure 16a. The areas with highest local values of ΔPE_z_ were found on the inner surface and near the ends of the reduction zone of the die. Values of ΔPE_z_ on the three featured layers are shown in Figure 16b to give better insight into the distribution of ΔPE_z_ along the axial direction. In the axial direction, a cyclic loading route on the inner surface can be found. The material is firstly compressed axially (with minus values) before the first contact with backward material flow. The material is mainly stretched axially in the reduction zone (I.I) and in the transition zone (I.II) it is compressed again. However, the middle and outer surface were only stretched. Two peaks (Peak 1 and Peak 2 in Figure 16b) could be seen in all curves at the beginning and end positions of the forging zone (I.I). Among all layers, the outer surface is of interest due to the direct contact to the die. By a high value of ΔPE_z_, a new surface is created on the workpiece and exposed to the die. The accompanying relative material flow under high normal stress could cause wear (for hard material) or adhesion (for soft material) on the dies. This could restrain the performance of the die and lower its lifetime and worsen the workpiece quality.

**Figure 17:**
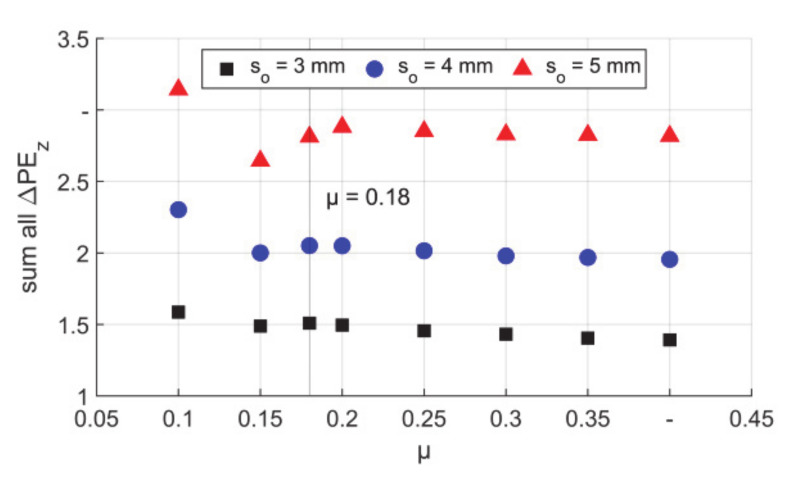
Sum of ΔPE_z_ between boundaries A and B.

In the deformation area, the sum of the values of ΔPE_z_ represents the overall axial elongation or compression, positive values for elongation, and negative values for compression. This value should reflect the plastic elongation in each stroke over the complete deformed area, see Figure 17. It has to be mentioned that the values of the curves for tubes with different wall thicknesses cannot be compared directly, as the values are dependent on the mesh numbers, for example, when one mesh is divided into two, there could be two meshes with the same value of ΔPE_z_, so the sum can be much higher. However, for all curves the same trend was found as it could be seen in Figure 11, which explained the plastic elongation by the material flow in one stroke. Therefore, a very smooth/lubricated contact pair (μ = 0.1) could result in the highest elongation in each stroke. However, it has to be mentioned that a considerable proportion of the elongation was caused by the backward material flow. In this situation, a deviation of the initial diameter and wall thickness was also generated, which was not beneficial for the precision.

**Figure 18:**
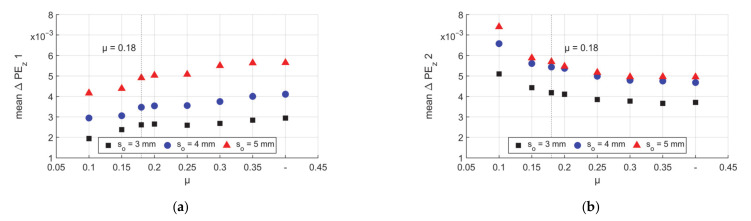
Mean values of axial ΔPE_z_ peaks, (**a**) Peak 1 and (**b**) Peak 2.

For the two peaks in Figure 16b, according to the method described in Figure 4, the mean height of the two peaks (Peak 1 and Peak 2) are shown in Figure 18a,b. For all tubes higher values of μ led to a higher mean value of Peak 1. This phenomenon can be explained as higher μ leads to less backward material flow on the first contact area (start in forging zone I.I), which leads to less negative values of ΔPE_z_ thus the trend increases. Oppositional, the height of Peak 2 decreases with higher μ as higher μ leads to higher friction resistance in the transition zone and calibration zone and leads to less local elongation. Combining Figure 18a,b it is seen that the location of both peaks on the die faces could be of risk of wear or adhesion depending on the friction value. As the values of mean ΔPE_z_ for Peak 1 are the highest for high µ and for Peak 2 the highest for low µ, and a friction coefficient between can spread the exposed new material on the contact pair, which could be better for the dies’ lifetime. However, it has to be mentioned that the wear of the die is also strongly dependent on the chemical condition, microstructure, and so on. When the process is lubricated by oil, the contact surface is cleaned and separated by the oil film, so there should be a relative trade–off, which leads to optimized material flow and low risk to the die life.

**Figure 19:**
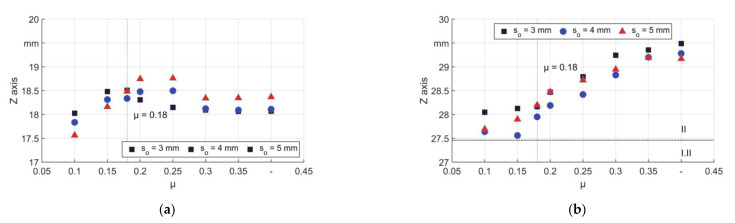
Barycenter of ΔPE_z_ peaks on the Z–axis, (**a**) Peak 1 and (**b**) Peak 2.

The locations of the barycenter of Peak 1 are in the reduction zone and near the first contact, see Figure 19a. As seen in Figure 12, low μ led to a higher outer diameter, which results in an earlier contact, so the barycenter features with low z coordinate. For all μ amounts higher than the self–locking condition, the diameter was nearly not increased so the first contact was later at a higher z coordinate. The barycenter moved inside the reduction zone for friction coefficients up to µ = 0.2 while a higher μ led to a backward movement of the barycenter. With a high friction resistance, the workpiece was held even tighter by the dies and with higher µ the material flow on the die surface was impeded, thus the barycenter moved a little backward.

The trend of Peak 2 in Figure 19b can also be explained by eliminated backward shifting after the self–locking condition, which enables more material flow towards the calibration zone. Higher μ led to less relative material flow on the contact area, and the material in transition was deformed more with local longest elongation. It should also be mentioned that the mesh was is not very small (0.33, 0.36, and 0.38 mm for s_0_ equals 3, 4, and 5 mm) compared with the changed locations of the barycenters. Thus, as introduced in Chapter 2, the locations need to be calculated by interpolation. Nevertheless, for μ higher than 0.1, the barycenters are always limited in small ranges. The first peak was in the range of around 0.5 mm. However, when μ equaled 0.1, the range was bigger (up to 1.2 mm for T5). The second peak for all the friction coefficients was in the range of 1.5 mm. The second peak was more sensitive to the friction condition than the first peak. Wear and adhesion were found on the locations of both peaks on the die [20].

### 3.3. Axial Plastic Strain Development

**Figure 20:**
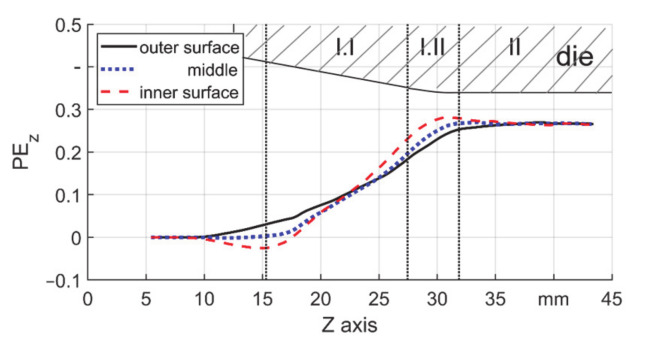
PE_z_ distribution of s_0_ = 3 mm and μ equals 0.18.

As the results of the accumulation of ΔPE_z_ in several strokes, the values of PE_z_ at different relative positions to the die show the deformation routes, see Figure 20. The simulation result with the self–locking friction coefficient is shown as an example. The other wall thickness and friction coefficients show similar features, but the curves with μ equaled 0.1 or 0.15 always started with negative values due to obvious backward material flow. It is seen that the inner surface was deformed with higher compression and stretching before the same final state in the calibration zone was reached. From the inner layer to the outer layer, the length of the deformation route reduced. These differences between each layer in the workpiece could be related to microstructure changes like the cold hardening or the final residual stress state. Furthermore, potential material failures like a wrinkle or crack could be identified.

**Figure 21:**
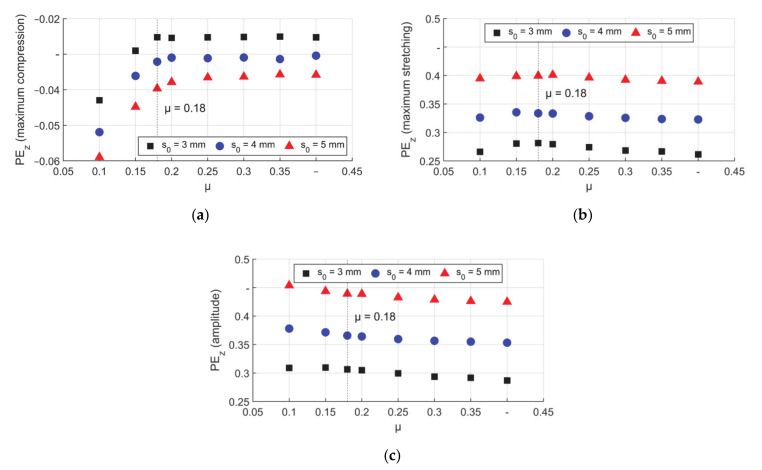
Peak values of axial PE_z_ on the inner surface, (**a**) maximum compression, (**b**) maximum stretching, and (**c**) amplitude.

The maximum axial compression on the inner surface was found at the beginning of the reduction zone and is shown in Figure 21a. As discussed above, low μ led to high axial compression, after self–locking the curve was nearly constant. The maximum stretching on the inner surface was found in the transition zone and is shown in Figure 21b. For low to high μ, the trend was a slow increase to a peak at the self–locking condition and then a slight decrease after that, which was more obvious for T3. This figure could be compared with the trend of the sum of ΔPE_z_ between boundaries A and B in Figure 17. The only difference for μ = 0.1 was that the backward material flow was high for this friction condition (starting with very low PE_z_ values in Figure 21a), thus the maximum stretching the material could reach at this position was limited. In Figure 21c it is shown that the amplitude of the plastic deformation for all the wall thicknesses decreased with increasing μ. For lower μ than self–locking condition, there was more axial compression in the start of the reduction zone. According to the combined hardening model, the yield stress will be lower once cyclic loading appeared. The higher the plastic strain is, the lower the yield stress after the reverse of loading direction will be. This inhomogeneous stress could lead to failure or lower precision of the product. A higher friction coefficient can reduce this inhomogeneity and further could reduce the risk.

**Figure 22:**
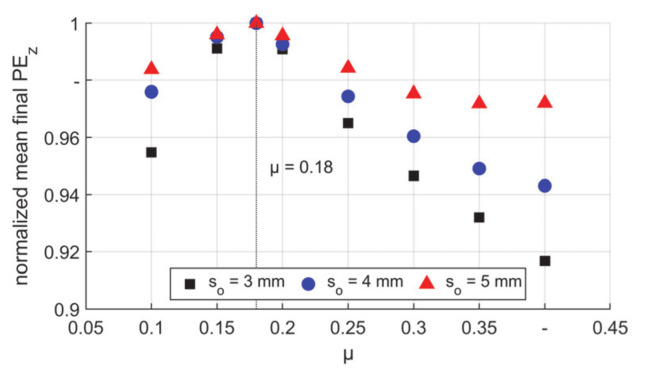
Final values of mean axial PE_z_, normalized values to μ = 0.18.

The final values of axial plastic strain component (PE_z_) were nearly the same regardless of the radial positions. In Figure 22 the final PE_z_ were normalized to the values of self–locking μ = 0.18. It is found that with the self–locking condition the maximum PE_z_ was achieved, which means the maximum elongation and consequently the thinnest wall after the process. The friction shows more influence on T3 that in this case, the difference of the final wall thickness caused by friction condition could reach up to 8.2% while for T5 it was only 2.8%. This could be explained by the increased radial volume of T5 in each stroke. Thus, the influence of friction coefficient reduced. Therefore, thinner tubes are more prone to wall thickness deviations caused by friction condition fluctuations.

## 4. Experimental Results and Validation

**Figure 23:**
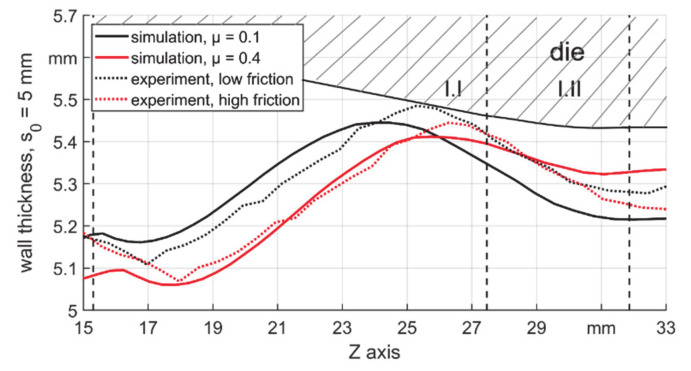
Wall thickness development of FE model and experiment with lowest and highest μ for s_0_ = 5 mm.

In this chapter, two findings from the FE model were validated. Firstly, the wall thickness development, especially in T5, was investigated. The results with the low (coated die with oil) and high (conventional die without oil) friction coefficients were compared in Figure 23. It could be seen that two regions with decreased wall thicknesses existed after the first contact and near the boundary between zones I.II and II, in both simulation and experiment results.

**Figure 24:**
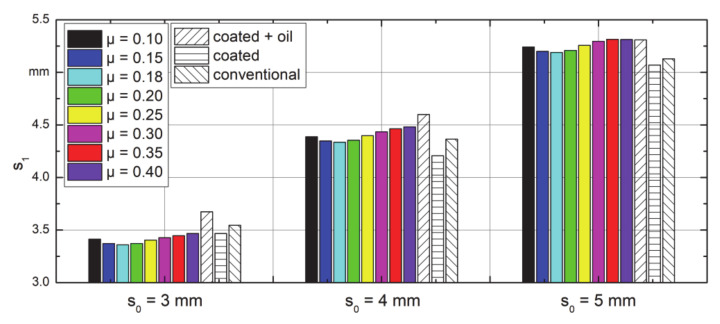
Wall thickness comparison between the FE model and experiments.

Further validation is done by the final axial plastic strain (PE_z_) in terms of wall thickness. The experimental measurement and those calculated from the FE model are shown in Figure 24. The trends of s_1_ are the same for all settings, which with increasing friction coefficient, firstly a decrease and then an increase can be observed. In the experiment, it was found that dry forming with coated dies led to the minimum final wall thickness, which means the longest final product. The maximum of the absolute value of deviation between the FE model and the experiment was less than 7%, so the FE model was well verified by the experiment. However, it is still notable that the results from the simulation were more similar with changing friction coefficients compared with that of the experiment. Additionally, the simulation results of different wall thicknesses show different accuracies, this could result from the deviation of the real experiments. In the reality, the friction condition was not ideal as the friction law, especially when oil was used, the process was much more complex than that in the simulation. The tube workpieces were made from rods, the wall thickness was controlled by the drill. In the experiment, anisotropic property of the workpiece material and the fluctuation (the feed per stroke and stroke height) of the rotary swaging system could also be a reason of deviation [28]. In the view of the FE model, the simplified 2D–asymmetric model and the rigid feeding strategy could also bring deviation. The former idealized tangential material flow and the latter makes the process more like a combination of rotary swaging and upsetting. Additionally, realizing different friction conditions on the three zones of the die should be considered. This is necessary to realize conventional dies in the FE model. They often feature an additionally roughened surface (high μ) in the reduction zone and a polished surface (lower μ) of the calibration zone.

## 5. Conclusions and Outlook

In this study, the material flow in steel tube workpieces with different initial wall thickness was investigated during the infeed rotary swaging process without a mandrel. The investigation was carried out in a 2D–axisymmetric FE model in ABAQUS. Compared with former research, the combined hardening material model was used in the FE model instead of the basic isotropic hardening model, thus the Bauschinger effect due to cyclic loading was taken into account. The simulation results were verified successfully by experiments in terms of wall thickness. A thorough insight into the back shifting, local geometry changing and detailed field of plastic strain components was realized. The influence of friction coefficient during the process on the product and the potential damage to the die was figured out. Which can help the industry for optimization. Following are the main findings:

(1) The material on the outer surface experiences radial cyclic deformation while the material on the inner surface experienced axial cyclic deformation. Thus, it is necessary to generate a combined hardening model;
(2) On the locations where redirected material flow appeared, the wall thickness tended to reduce due to stress concentration. This phenomenon was more obvious in thick tubes;
(3) To reduce back shifting and backward material flow, the friction coefficient on the contact zone should be even higher than the self–locking value;
(4) The material near the inner surface was deformed with higher amplitude of axial plastic strain, which could be reduced by increasing friction coefficient. This reduced amplitude could lead to a more homogeneous deformation in the workpiece;
(5) The self–locking friction condition enabled maximum elongation of the workpiece thus the thinnest wall after the process.

According to the findings, further experiments were needed to figure out the mechanism of material flow affected by different processing parameters like feed per stroke. Additionally, in the simulation, the complete contact area was simplified with the same friction coefficient, but in reality, the reduction zone, transition zone, and calibration zone could be applied with a different structure or coatings to realize different friction conditions This means an even more complex friction condition, following research could take these issues into account.

## Figures and Tables

**Table 1:** Parameters of nonlinear isotropic/kinematic hardening model of steel alloy 1.0308.

σ_s_ (mPa)	Q (mPa)	b	C_1_	γ_1_	C_2_	γ_2_	C_3_	γ_3_
170.50	168.16	14.82	1614.1	41.947	1543.2	40.704	239.00	0

## Data Availability

The data presented in this study are available on request from the corresponding author.
